# Numerical Simulations Based on a Meshfree Method for Nickel-Steel Welded Joint Manufactured by Micro-Jet Cooling

**DOI:** 10.3390/ma15238579

**Published:** 2022-12-01

**Authors:** Anita Uściłowska, Bożena Szczucka-Lasota, Tomasz Węgrzyn, Tadeusz Szymczak, Bogusław Łazarz, Joanna Kamińska

**Affiliations:** 1 Faculty of Mechanical Engineering, Institute of Materials Technology, Poznan University of Technology, 60-965 Poznan, Poland; 2 Department of Road Transport, Faculty of Transport and Aviation Engineering, Silesian University of Technology, 44-100 Gliwice, Poland; 3 Department of Automotive Vehicle Maintenance, Faculty of Transport and Aviation Engineering, Silesian University of Technology, 44-100 Gliwice, Poland; 4 Department of Vehicle Type-Approval & Testing, Motor Transport Institute, 03-301 Warsaw, Poland; 5 Department of Applied Mathematics, The Faculty of Environmental Engineering and Geodesy, Wroclaw University of Environmental Life Sciences, 50-357 Wroclaw, Poland

**Keywords:** energy devices, superalloy, hard-rusting steel, method of fundamental solutions, welding, micro-jet cooling, mechanical engineering

## Abstract

The article presents a numerical–experimental approach to the weldability and mechanical resistance of the joint of Alloy 59 (2.4605, nickel-chromium-molybdenum) and S355J2W (1.8965) structural steel manufactured by the MIG process with the use of micro-jet cooling. This research was considered because the standard MIG process does not guarantee the procurement of a mixed hard-rusting structural steel superalloy weld of a repeatable and acceptable quality. Welds made through the classic MIG process express cracks that result from their unfavorable metallographic microstructure, while the joint supported by micro-jet cooling does not reflect any cracks and has a high strength with good flexibility. This was achieved by the application of helium for cooling. The joining technology was also considered in the numerical stage, represented by calculations in situ. For this purpose, the fundamental solution method (FSM) for the simulation of heat transfer during the process of welding with micro-jet cooling was implemented according to the initial boundary value problem (IBVP). The problem was solved employing the method of combining the finite difference method, Picard iterations, approximation by the radial basis function, and the fundamental solution method so as to solve the IVBP. The proposed method was validated by the data and results obtained during in situ experiments. The numerical approach enabled us to obtain variations in the temperature distribution values in HAZ with its different dimensional variants, ranging between 600 °C and 1400 °C.

## 1. Introduction

Mixed steel-nickel alloy joints have become more prevalent in civil engineering in light of the technological features of the components. Inconel-grade nickel alloys (nickel alloys with chromium, molybdenum, and iron) are highly weldable in shielding gases when utilizing conventional welding technologies, such as MIG (metal inert gas) and TIG (tungsten inner gas). Their good weldability is a result of their favorable austenitic microstructure, guaranteeing their acceptable ductility.

The Alloy 59 (NiCr23Mo16Al) is a superalloy used in structures intended for operations at elevated and high temperatures, as well as in aggressive environments [1,2]. This kind of material is particularly resistant to pitting and crevice corrosion [3] and, therefore, it has also found the following applications: the shipbuilding and aviation industries, as well as pollution control devices and nuclear reactors [4,5]. 

The S355 steel grade is a structural material that is widely used in various branches of industry, such as automotive and power plants. The significant advantage of the material is related to its excellent weldability and yield stress (355 MPa), as well as its high level of flexibility [6], which eliminates brittle cracking. Moreover, the S355J2 steel is not considered highly weldable due to the frequent occurrence of hot cracks [7]. For this reason, it is recommended to preheat this steel to the temperature of 100 °C before welding [8]. The type of selected shielding gas mixtures [9] may also affect the formation of hot cracks. The best results are achieved when welding hard-rusting steel through the MAG and MIG processes [9]. 

One should note that it is difficult to achieve a correctly mixed joint (Inconel-grade material with hard-rusting steel), especially for materials over 4 mm in thickness, due to the formation of cracks [10]. An additional disadvantage is related to the different values of the thermal conductivity coefficients of both materials. Inconel and hard-rusting steel also have different linear and volumetric expansion coefficients, which is unfavorable when trying to obtain the correct joint [11]. The linear expansion coefficient of a superalloy is at the level of 13.1 × 10^−6^ 1/K, and that of steel resistant to rust is 10.3 × 10^−6^ 1/K. The expansion and volumetric coefficient of Inconel are at the level of 39.1 × 10^−6^ 1/K, and that of hard-rusting steel is 32.2 × 10^−6^ 1/K. The welding of joints made of hard-rusting steel-superalloy is not often analyzed in the literature. Theoretical considerations on this topic were presented by Ferreira A.F. [12] and others [13,14,15], formulating the relationship between the micro-segregation, phases, and dendritic grain growth of hypoeutectic alloys.

To explore further details on the microstructure of an alloy containing nickel, iron, and chromium (assuming molybdenum is the equivalent of chromium, according to the Schaeffler diagram) [16,17,18], the triple equilibrium system of Fe-Ni-Cr should be analyzed [19]. Joints, in which a columnar microstructure is formed during solidification, have the most remarkable tendency to fracture [15,20]. The cracking of these joints is promoted by their relatively smooth grain boundary surfaces [21]. Rajasekhar and Harendranath indicated that a dendritic cell microstructure should be formed during the solidification of the joint to avoid cracks in the joint [13].

The method of fundamental solutions (MFS, [22]) is one of the so-called meshfree methods and was proposed by Kupradze and Aleksidze in [23]. The authors of [24] presented the first numerical implementation of MFS. One of the papers considering the numerical background of the use of MFS to solve non-linear boundary value problems (BVPs) is [25]. The authors proposed implementing the analog equation method (AEM) to solve non-linear BVPs. MFS and approximation by the radial basis function (RBF) have been implemented to solve inhomogeneous equations. 

The authors of [26] used the algorithm which combines the MFS and the asymptotic numerical method (ANM) to solve two-dimensional non-linear elastic problems. Using the Taylor series, the non-linear modifiable problem was transformed into a series of linear differential equations with the same tangent operator. The method of fundamental solutions–radial basis function (MFS-RBF) was combined with the AEM to solve these resulting linear equations. Regularization methods such as truncated singular value decomposition (TSVD) and Tikhonov regularization were used to control the resulting ill-conditioned linear systems. 

The other approach for solving non-linear problems is the application of the homotopy analysis method (HAM) combined with MFS [27]. The HAM was introduced to linearize equations in an analytical way. The BVP given by non-linear equations with linear boundary conditions is transformed into a set of BVPs given by linear equations with modified linear boundary conditions. The authors of [28] considered solving the BVP using a nonlinear equation with non-linear boundary conditions. They transformed the non-linear governing partial differential equation into the Laplace equation with non-linear boundary conditions by applying the Kirchhoff transformation. 

Another class of methods combining MFS with other procedures are those used to solve transient problems (time-dependent ones). The implementation of MFS combined with the finite difference method (FDM) for solving transient BVPs with the chosen type of parabolic equation was proposed in [29]. The authors indicated that the Laplace transform can be used to solve the time-dependent problem. Moreover, to address the numerical instability of the approach, they proposed applying FDM for the approximation of the differential variable with respect to the time variable. They solved the Helmholtz-type equations each time using MFS-RBF. 

In [30], the implementation of MFS for the transient temperature distribution is presented. The Laplace transform, combined with MFS, was discussed. Then, the performance of the finite difference method (FDM) combined with MFS was shown. The phenomenon of transient heat transfer was considered in [31] using a very general approach. The modified MFS was implemented to simulate such problems. This version of MFS (with the source point placed in the considered region) is also a perfect tool for solving transient issues. Moreover, the method of the fundamental solution was extended to the condition of moving boundaries [32]. The proposed innovation of MFS was implemented to solve the problem of melting the material. One of the examples of a non-linear problem was presented in [1]. The application of the method of fundamental solutions to two-dimensional steady-state heat conduction problems for both isotropic and anisotropic, single and composite (bi-materials), and non-linear, functionally graded materials (FGMs) was presented. The authors applied the Kirchhoff transform and obtained homogeneous equations of which the fundamental solution is known. The other problem of heat conduction was considered in [33]. The author focused on the minimal solution to the steady-state blow-up problem. The numerical method based on the approach of fundamental solutions, thin-plate spline interpolation, and monotone iteration was proposed and applied.

The nonlinear 3D problem of magnetostatics was solved by the MFS supported by an iterative algorithm [34]. The authors summarized the obtained results, and they were found to be prospective. 

Some non-linear and transient biomechanics problems were solved using numerical methods based on MFS [35]. A 2D non-linear skin model with a temperature-dependent blood perfusion rate was studied. The bioheat transfer equation with non-linear terms induced by the temperature-dependent blood perfusion rate was linearized using Taylor’s expansion technique. Then, the authors proposed solving the linearized governing equation with given boundary conditions by applying the dual reciprocity method and the MFS. Several numerical examples involving linear, quadratic, and exponential relations between the temperature and blood perfusion rate were tested to verify the efficiency and accuracy of the proposed meshless numerical procedure. The other non-linear problem is related to the flow in a porous medium with a free surface. The 3D version of such a flow was considered in [36]. The problem is the BVP with the Laplace equation and non-linear boundary conditions. The nonlinearity of the free surface flow is rooted in the boundary conditions of the moving surface. In the study, the relaxation method was applied to linearize the problem. 

Therefore, two fundamental aims of the paper are formulated, i.e., the theoretical and practical. In regard to the first indicated approach, the implementation of MFS with other additional methods for solving transient heat transfer in welded different materials (with different thermal characteristics, i.e., the weld, heat-affected zone (HAZ), and base metal (BM)) with non-linear thermal characteristics was selected. The finite difference method was applied to approximate the time derivative, and Picard iterations were used to treat the nonlinearities of the equations and boundary conditions. The system of equations with boundary conditions was solved using MFS-RBF combinations. Such an approach is entirely new in the literature, and it concerns a multi-material domain with nonlinearities originating from different materials. In this case, the thermal phenomena occurring in the mixed joint were detailed, considering the other material properties of both welded components and using the method of fundamental solutions.

The practical aspect of the paper aims to provide the most suitable technology and its parameters for the manufacturing of a mixed joint with the best possible microstructure and mechanical properties. Therefore, we decided to verify the welding potential of a mixed joint made of Alloy 59 (nickel-chromium-molybdenum) and the non-alloy structural steel S355J2W using the MIG process, as well as various micro-jet cooling parameters. The presented results provided the basis for our conclusions regarding the selection of parameters to produce a mixed butt welded joint. Once the mixed joint was prepared with the use of the classic MIG process and the modified MIG process (with the application of micro-jet cooling), the microstructure and properties of the joint were determined. 

## 2. Details on the Material and Welding Method

The selected materials were mixed welded joints made using the MIG process (131). The joints were made from 8 mm-thick sheets. The mechanical properties of the S355J2 steel in the as-received state, Alloy 59 [37], and the NiCr23Mo16 electrode wire ( Böhler, Germany,) weld metal (according to the EN ISO 18274 standard) are presented in Table 1. 

The mentioned welding wire (superalloy from the alloy group 59) is used in highly aggressive environments. It is recommended for the joining of duplex and super duplex steels, stainless steels, and various nickel-based alloys [38]. Based on the data presented in Table 1, significant differences in the ultimate tensile strength and yield stress values of both joined materials can be concluded. The given properties result from the different chemical compositions of the used materials. Table 2 shows the chemical compositions of the S355J2 steel, Alloy 59, and weld metal of the electrode wire.

Due to the purpose of the use of the materials outlined above and subjected to an extensive range of temperatures during welding, the thermal conductivity coefficient and specific heat capacity must be treated as a temperature function. The proper characteristics are given below:

(1) $$\begin{array}{ll} {\tilde{c}}_{p,Ni}\left(T\right)= & -5.739462\cdot 10^{-10}T^{4}+1.59927\cdot 10^{-6}T^{3}\\ & -1.404298\cdot 10^{-3}T^{2}+4.915666\cdot 10^{-1}T+40.95882 \end{array}$$

(2) $${\tilde{c}}_{p,St}\left(T\right)=\{\begin{array}{rr} \begin{array}{r} 6.784588\cdot 10^{-9}T^{4}-8.202465\cdot 10^{-6}T^{3}+3.415793\cdot 10^{-3}T^{2}\\ -1.193297\cdot 10^{-1}T+455.3738 \end{array} & T<750\\ \begin{array}{r} 2.703963\cdot 10^{-8}T^{4}-1.48925\cdot 10^{-4}T^{3}+3.064794\cdot 10^{-1}T^{2}\\ -2.794481\cdot 10^{2}T+95944.12 \end{array} & T>750 \end{array}$$

(3) $${\tilde{c}}_{p,w}\left(T\right)=9.920635\cdot 10^{-9}T^{4}-2.075992\cdot 10^{-5}T^{3}+1.554281\cdot 10^{-2}T^{2}-4.649049t+891,8942$$

(4) $${\tilde{\lambda }}_{Ni}\left(T\right)=1.287139\cdot 10^{-5}T^{2}-3.6962\cdot 10^{-4}T+12.70055$$

(5) $$\begin{array}{ll} {\tilde{\lambda }}_{St}\left(T\right)= & -1.682147\cdot 10^{-10}T^{4}+5.495677\cdot 10^{-7}T^{3}-6.105904\cdot 10^{-4}T^{2}\\ & +2.542832\cdot 10^{-1}T+2.631845 \end{array}$$

(6) $${\tilde{\lambda }}_{w}\left(T\right)=1.672124\cdot 10^{-5}T^{2}+1.212307\cdot 10^{-3}T+10.16585$$

where ${\tilde{\lambda }}_{Ni}\left(T\right)$, ${\tilde{\lambda }}_{St}\left(T\right)$, and ${\tilde{\lambda }}_{w}\left(T\right)$ describe the thermal conductivity coefficient (W/(m·K)), ${\tilde{c}}_{p,Ni}\left(T\right)$, ${\tilde{c}}_{p,Sti}\left(T\right)$, and ${\tilde{c}}_{p,w}\left(T\right)$ are the specific heat capacity (J/(kg·K)), and *T* denotes the temperature field (K). The subscripts mean the following: *Ni*—Inconel alloy, *St*—steel alloy, and *w*—weld.

**Figure 1:**
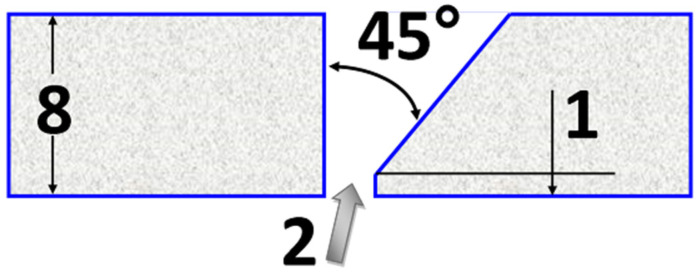
Groove shape, dimensions in [mm].

Concerning the different thermal conductivity coefficients of the two welded alloys, we decided to prepare joints with one-sided bevels of 1/2 V. The throat angle was at the level of 45°. The beveling method of the steel sheet is presented in Figure 1.

**Figure 2:**
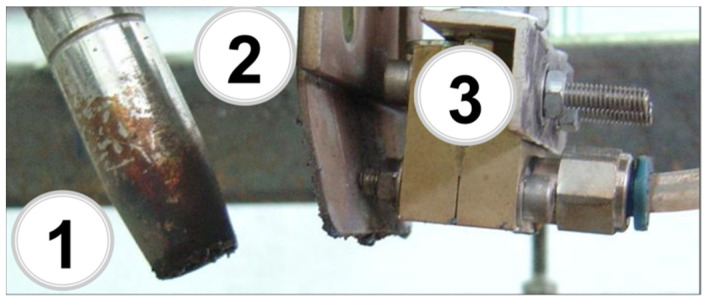
Assembly of the welding head and micro-jet injector: 1—welding head, 2—handle, 3—injector.

The welding parameters of the joint were as follows: the electrode wire diameter was 1.2 mm, the arc voltage U = 21 V, and the welding current intensity was at the level of 137 A (Figure 2). The welded sheets had the dimensions of 800 × 200 × 8 mm. Argon was selected to act as a shielding gas in the MIG process. The shielding gas flow rate was at the level of 15 l/min. The welding speed during the laying of all three stitches was 240 mm/min. The MIG welding method (131) in the downward position (PA) was used according to the requirements of the EN 15614-1 standard. The rotor joints were welded with a direct current with a positive polarity of the electrode.

In this case, preheating was not used. Micro-jet cooling was applied directly after the welding. The weld metal deposit was prepared using the MIG welding method with and without micro-jet cooling. Helium was selected as a micro-jet gas. The main parameters of the micro-jet cooling varied slightly, including the cooling steam diameter (50 µm and 60 µm) and gas pressure (0.4 MPa and 0.5 MPa).

**Figure 3:**
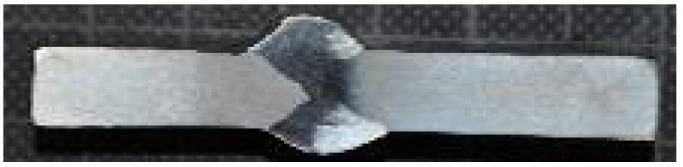
Macroscopic photo of an 8 mm-thick joint made of S355J2 hard-rusting steel (left part of the connection) with Alloy 59 (right part of the connection).

The assembly of the welding head and the micro-jet injector is illustrated in Figure 2. One of the obtained joints is presented in Figure 3.

## 3. Numerical Approach

The method of fundamental solutions is applied to simulate the heat transfer process during the welding of two types of metals. The phenomenon is modeled as an initial-boundary value problem, which is described by the system of non-linear partial differential equations with initial conditions and non-linear boundary conditions. The nonlinearity results from the non-linear thermal characteristics of the welded materials. The thermal conductivity and heat capacity of the superalloy and steel are described by the nonlinear function of the temperature. It is necessary to consider these nonlinearities due to the range of temperatures in the welding and cooling process.

### 3.1. Problem Description and Mathematical Model

**Figure 4:**
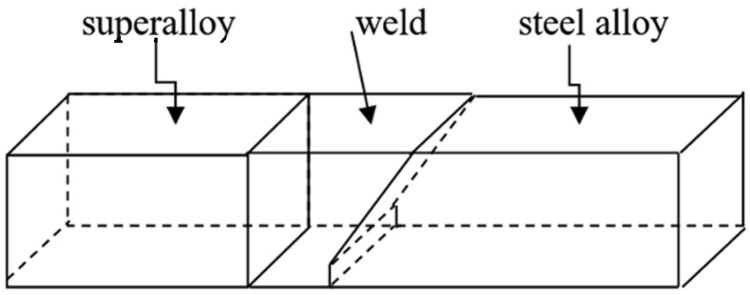
Geometry of the welded materials.

**Figure 5:**
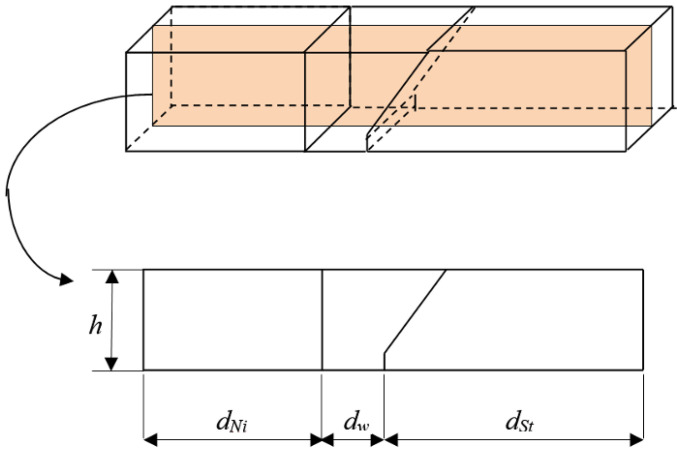
Cross-section of the welded materials.

The transient temperature is considered in the case of the welded materials. A scheme of the welded objects is presented in Figure 4. There are three regions: the weld with cooling and the welded materials, the superalloy and steel. The cross-section shown in Figure 5 is taken into account.

The geometry of these regions is described as follows. An area occupied by the superalloy is defined as ${\tilde{\Omega }}_{Ni}$, the steel alloy is ${\tilde{\Omega }}_{St}$, and the area occupied by the weld is ${\tilde{\Omega }}_{w}$, where *d_Ni_* is the length of the horizontal rectangle edge of the area ${\tilde{\Omega }}_{Ni}$, $\tilde{h}$ is the thickness of the plate, *d_w_* is the width of the weld, and *d_st_* is the length of the lower horizontal edge of the steel zone.

**Figure 6:**
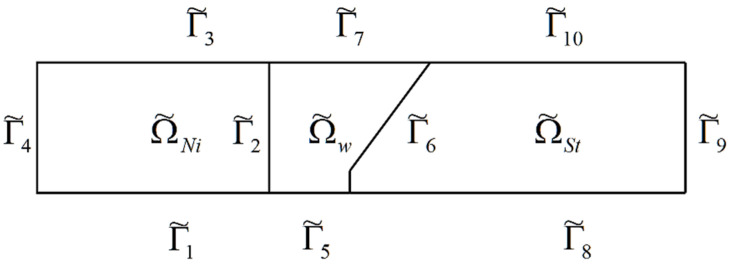
Geometry: nomenclature of areas and boundaries.

The boundaries of the regions are indicated in Figure 6, and the boundary of the superalloy is ${\tilde{\Gamma }}_{Ni}={\tilde{\Gamma }}_{1}\cup {\tilde{\Gamma }}_{2}\cup {\tilde{\Gamma }}_{3}\cup {\tilde{\Gamma }}_{4}$, the boundary of the steel alloy is ${\tilde{\Gamma }}_{St}={\tilde{\Gamma }}_{8}\cup {\tilde{\Gamma }}_{9}\cup {\tilde{\Gamma }}_{10}\cup {\tilde{\Gamma }}_{6}$, and the boundary of the weld is ${\tilde{\Gamma }}_{w}={\tilde{\Gamma }}_{5}\cup {\tilde{\Gamma }}_{6}\cup {\tilde{\Gamma }}_{7}\cup {\tilde{\Gamma }}_{2}$.

The equations of the transient heat transfer are introduced for each region under consideration. Thus, the equations are in the forms:

- For the superalloy:

(7) $${\tilde{c}}_{p,Ni}\left(t_{Ni}\right){\tilde{\rho }}_{Ni}\frac{\partial t_{Ni}}{\partial t}=\nabla \cdot \left({\tilde{\lambda }}_{Ni}\left(t_{Ni}\right)\nabla t_{Ni}\right)\;\mathrm{for}\;\left(\tilde{x},\tilde{y}\right)\in {\tilde{\Omega }}_{Ni}$$

- For the steel alloy:

(8) $${\tilde{c}}_{p,St}\left(t_{St}\right){\tilde{\rho }}_{St}\frac{\partial t_{St}}{\partial t}=\nabla \cdot \left({\tilde{\lambda }}_{St}\left(t_{St}\right)\nabla t_{St}\right)\;\mathrm{for}\;\left(\tilde{x},\tilde{y}\right)\in {\tilde{\Omega }}_{St}$$

- For the weld:

(9) $${\tilde{c}}_{p,w}\left(t_{w}\right){\tilde{\rho }}_{w}\frac{\partial t_{w}}{\partial t}=\nabla \cdot \left({\tilde{\lambda }}_{w}\left(t_{w}\right)\nabla t_{w}\right)\;\mathrm{for}\;\left(\tilde{x},\tilde{y}\right)\in {\tilde{\Omega }}_{w}$$

where *t* denotes time, and $\tilde{x},\tilde{y}$ are geometrical coordinates. The other terminology is adequate for each considered area, noted by subscripts: *Ni*—for the superalloy, *St*—for the steel alloy, and *w*—for the weld. Additionally, the terminology is as follows: $t_{Ni}$, $t_{St}$, $t_{w}$—temperature in the areas, ${\tilde{\rho }}_{Ni}$, ${\tilde{\rho }}_{St}$, ${\tilde{\rho }}_{w}$—mass density, ${\tilde{c}}_{p,Ni}\left(t_{Ni}\right)$, ${\tilde{c}}_{p,St}\left(t_{St}\right)$, ${\tilde{c}}_{p,w}\left(t_{w}\right)$—specific heat capacity, and ${\tilde{\lambda }}_{Ni}\left(t_{Ni}\right)$, ${\tilde{\lambda }}_{St}\left(t_{St}\right)$, ${\tilde{\lambda }}_{w}\left(t_{w}\right)$—thermal conductivity coefficient. The values of the physical parameters applied in the numerical approach at the areas examined are listed in Equations (1)–(6).

The problem is defined for the chosen time interval, i.e., $t\in \left[t_{0},\infty \right)$, and $t_{0}$ is the initial time. The associated initial conditions describe the initial temperature of the superalloy and steel alloys, which is the ambient temperature:

(10) $$t_{Ni}\left(\tilde{x},t_{0}\right)={\tilde{f}}_{Ni}\left(\tilde{x},\tilde{y}\right)$$

(11) $$t_{St}\left(\tilde{x},t_{0}\right)={\tilde{f}}_{St}\left(\tilde{x},\tilde{y}\right)$$

and the temperature of weld, which is the temperature of the welding:

(12) $$t_{w}\left(\tilde{x},t_{0}\right)={\tilde{f}}_{w}\left(\tilde{x},\tilde{y}\right)$$

where $\tilde{x}=\left(\tilde{x},\tilde{y}\right)$, ${\tilde{f}}_{Ni}\left(\tilde{x},\tilde{y}\right)$ describes the initial temperature of the superalloy, ${\tilde{f}}_{St}\left(\tilde{x},\tilde{y}\right)$ describes the initial temperature of the steel alloy, and ${\tilde{f}}_{w}\left(\tilde{x},\tilde{y}\right)$ describes the initial temperature of the welding element.

The problem is determined by the boundary conditions. The free surfaces of the superalloy-steel alloys are subjected to natural convection:

(13) $${\tilde{\lambda }}_{Ni}\left(t_{Ni}\right)\frac{\partial t_{Ni}}{\partial \tilde{n}}={\tilde{a}}_{Ni}\left(t_{Ni}-t_{am}\right)\;\mathrm{on}\;{\tilde{\Gamma }}_{1}\cup {\tilde{\Gamma }}_{3}$$

(14) $${\tilde{\lambda }}_{St}\left(t_{St}\right)\frac{\partial t_{St}}{\partial \tilde{n}}={\tilde{a}}_{St}\left(t_{St}-t_{am}\right)\mathrm{on}\;{\tilde{\Gamma }}_{8}\cup {\tilde{\Gamma }}_{10}$$

where ${\tilde{a}}_{Ni}$ and ${\tilde{a}}_{St}$ are the superalloy and steel convection coefficients, respectively, and *t_am_* is the ambient temperature.

Regarding the boundaries ${\tilde{\Gamma }}_{4}$ and ${\tilde{\Gamma }}_{9}$, it is assumed that the boundaries are far from the HAZ, and there is a constant temperature equal to the ambient temperature:

(15) $$t_{Ni}=t_{am}\;\mathrm{on}\;{\tilde{\Gamma }}_{4}$$

(16) $$t_{St}=t_{am}\;\mathrm{on}\;{\tilde{\Gamma }}_{9}$$

On the outer boundaries of the weld, the cooling process is applied. Thus, the boundary condition is given by the function, which describes the cooling as follows:

(17) $$t_{w}=f_{w,d}\left(\hat{x},t\right)\mathrm{on}\;{\tilde{\Gamma }}_{5}$$

(18) $$t_{w}=f_{w,u}\left(\hat{x},t\right)\mathrm{on}\;{\tilde{\Gamma }}_{7}$$

It is assumed that there is perfect contact between the contacted regions. This means that the following boundary conditions should be fulfilled:

(19) $$t_{Ni}=t_{w}\;\mathrm{on}\;{\tilde{\Gamma }}_{2}$$

(20) $${\tilde{\lambda }}_{Ni}\left(t_{Ni}\right)\frac{\partial t_{Ni}}{\partial \tilde{n}}={\tilde{\lambda }}_{w}\left(t_{w}\right)\frac{\partial t_{w}}{\partial \tilde{n}}\;\mathrm{on}\;{\tilde{\Gamma }}_{2}$$

(21) $$t_{St}=t_{w}\;\mathrm{on}\;{\tilde{\Gamma }}_{6}$$

(22) $${\tilde{\lambda }}_{St}\left(t_{St}\right)\frac{\partial t_{St}}{\partial \tilde{n}}={\tilde{\lambda }}_{w}\left(t_{w}\right)\frac{\partial t_{w}}{\partial \tilde{n}}\;\mathrm{on}\;{\tilde{\Gamma }}_{6}$$

where ${\tilde{\lambda }}_{Ni}\left(t_{Ni}\right)$, ${\tilde{\lambda }}_{St}\left(t_{St}\right)$, and ${\tilde{\lambda }}_{w}\left(t_{w}\right)$ are the thermal conductivity of the superalloy, steel, and weld, respectively.

Due to the range of temperatures that appear in the welding process, the material characteristics, such as the specific heat capacity and thermal conductivity coefficient, for every material used are described by Equations (1)–(6).

To avoid numerical errors, it is convenient to consider the problem in a non-dimensional form. The dimensionless variables are introduced as follows:

(23) $$x=\frac{\tilde{x}}{L},\;y=\frac{\tilde{y}}{L},\;h=\frac{\tilde{h}}{L},\;d=\frac{\tilde{d}}{L}$$

$$T_{Ni}=\frac{t_{Ni}-t_{am}}{t_{\max}-t_{am}},\;T_{St}=\frac{t_{St}-t_{am}}{t_{\max}-t_{am}},\;T_{w}=\frac{t_{w}-t_{am}}{t_{\max}-t_{am}}$$

$$c_{p,Ni}\left(t_{Ni}\right)=\frac{{\tilde{c}}_{p,Ni}\left(t_{Ni}\right)}{c_{p,0}},\;c_{p,t}\left(t_{St}\right)=\frac{{\tilde{c}}_{p,St}\left(t_{St}\right)}{c_{p,0}},\;c_{p,w}\left(t_{w}\right)=\frac{{\tilde{c}}_{p,w}\left(t_{w}\right)}{c_{p,0}}$$

$$\lambda_{Ni}\left(t_{Ni}\right)=\frac{{\tilde{\lambda }}_{Ni}\left(t_{Ni}\right)}{\lambda_{0}},\lambda_{St}\left(t_{St}\right)=\frac{{\tilde{\lambda }}_{St}\left(t_{St}\right)}{\lambda_{0}},\;\lambda_{w}\left(t_{w}\right)=\frac{{\tilde{\lambda }}_{w}\left(t_{w}\right)}{\lambda_{0}}$$

where *t*_max_ is the maximum temperature of the weld, *t_am_* is the ambient temperature, $\lambda_{0}$ is the reference thermal conductivity coefficient, and $c_{p,0}$ is the reference specific heat capacity.

The area given in the dimensionless form and occupied by the superalloy is $\Omega_{Ni}$, the steel alloy is $\Omega_{St}$, and the dimensionless area occupied by the weld is $\Omega_{w}$. In the dimensionless form, the boundaries of the areas are as follows: $\Gamma_{1}$, $\Gamma_{2}$, $\Gamma_{3}$, $\Gamma_{4}$, $\Gamma_{5}$, $\Gamma_{6}$,$\Gamma_{7}$, $\Gamma_{8}$, $\Gamma_{9}$, $\Gamma_{10}$.

Therefore, the boundary of the region $\Omega_{Ni}$ is $\Gamma_{Ni}=\Gamma_{1}\cup \Gamma_{2}\cup \Gamma_{3}\cup \Gamma_{4}$, the boundary of the region $\Omega_{St}$ is $\Gamma_{St}=\Gamma_{8}\cup \Gamma_{9}\cup \Gamma_{10}\cup \Gamma_{6}$, and the boundary of the region $\Omega_{w}$ is described as $\Gamma_{w}=\Gamma_{5}\cup \Gamma_{6}\cup \Gamma_{7}\cup \Gamma_{2}$.

Then, the governing Equations (7)–(9) have the dimensionless form in the superalloy, steel, and the weld areas:

(24) $$c_{p,Ni}\left(T_{Ni}\right)\rho_{Ni}\left(T_{Ni}\right)\frac{\partial T_{Ni}}{\partial \tau }=\nabla \cdot \left(\lambda_{Ni}\left(T_{Ni}\right)\nabla T_{Ni}\right)\;\mathrm{for}\;\left(x,y\right)\in \Omega_{Ni}$$

(25) $$c_{p,St}\left(T_{St}\right)\rho_{St}\left(T_{St}\right)\frac{\partial T_{St}}{\partial \tau }=\nabla \cdot \left(\lambda_{St}\left(T_{St}\right)\nabla T_{St}\right)\;\mathrm{for}\;\left(x,y\right)\in \Omega_{St}$$

(26) $$c_{p,w}\left(T_{w}\right)\rho_{w}\left(T_{w}\right)\frac{\partial T_{w}}{\partial \tau }=\nabla \cdot \left(\lambda_{w}\left(T_{w}\right)\nabla T_{w}\right)\;\mathrm{for}\;\left(x,y\right)\in \Omega_{w}$$

where the dimensionless forms of the specific heat capacity and thermal conductivities are: $c_{p,Ni}\left(T_{Ni}\right)$, $c_{p,St}\left(T_{St}\right)$, $c_{p,w}\left(T_{w}\right)$, $\lambda_{Ni}\left(T_{Ni}\right)$, $\lambda_{St}\left(T_{St}\right)$, and $\lambda_{w}\left(T_{w}\right)$.

The initial conditions have the following form.

The associated initial conditions describe the dimensionless initial temperature of the superalloy and steel alloys, which is the ambient temperature (in the dimensionless form):

(27) $$T_{Ni}\left(x,\tau_{0}\right)=f_{Ni}\left(x,y\right)$$

(28) $$T_{St}\left(x,\tau_{0}\right)=f_{St}\left(x,y\right)$$

And the temperature of the welding:

(29) $$T_{w}\left(x,\tau_{0}\right)=f_{w}\left(x,y\right)$$

where x=(x,y) and $\tau_{0}$ is the initial non-dimensional time.

The boundary conditions have the following dimensionless form:

(30) $$\lambda_{Ni}\left(T_{Ni}\right)\frac{\partial T_{Ni}}{\partial n}=a_{Ni}\left(T_{Ni}-T_{am}\right)\;\mathrm{on}\;\Gamma_{1}\cup \Gamma_{3}$$

(31) $$\lambda_{St}\left(T_{St}\right)\frac{\partial T_{St}}{\partial n}=a_{St}\left(T_{St}-T_{am}\right)\mathrm{on}\;\Gamma_{8}\cup \Gamma_{10}$$

(32) $$T_{Ni}=T_{am}\;\mathrm{on}\;\Gamma_{4}$$

(33) $$T_{St}=T_{am}\;\mathrm{on}\;\Gamma_{9}$$

(34) $$T_{w}=f_{w,d}\left(x,\tau \right)\mathrm{on}\;\Gamma_{5}$$

(35) $$T_{w}=f_{w,u}\left(x,\tau \right)\mathrm{on}\;\Gamma_{7}$$

(36) $$T_{Ni}=T_{w}\;\mathrm{on}\;\Gamma_{2}$$

(37) $$\lambda_{Ni}\left(T_{Ni}\right)\frac{\partial T_{Ni}}{\partial n}=\lambda_{w}\left(T_{w}\right)\frac{\partial T_{w}}{\partial n}\;\mathrm{on}\;\Gamma_{2}$$

(38) $$T_{St}=T_{w}\;\mathrm{on}\;\Gamma_{6}$$

(39) $$\lambda_{St}\left(T_{St}\right)\frac{\partial T_{St}}{\partial n}=\lambda_{w}\left(T_{w}\right)\frac{\partial T_{w}}{\partial n}\;\mathrm{on}\;\Gamma_{6}$$

To solve the initial boundary problem given by Equations (24)–(39), the numerical algorithm is proposed.

### 3.2. Numerical Procedure

The main assumption for the numerical procedure is that one must apply one of the mesh-free methods: the method of fundamental solutions (MFS). However, for the initial problem, an auxiliary method should be used to treat the differential temperature function according to time. This paper proposes approximating the differential concerning the time by the finite difference (FD). To implement the FD, the time interval, $\tau \in \left[\tau_{0},\tau_{\max}\right]$, in which the solution is sought, is divided into *n_t_* subintervals. The solutions are obtained in certain time steps:

$$\tau_{i}=i\cdot d\tau$$

where $d\tau =\frac{\tau_{\max}-\tau_{0}}{n_{t}}$ and *i* = 1, …, $n_{t}$.

The partial derivative with respect to time is approximated by:

(40) $${\frac{\partial T_{Ni}\left(x,y,\tau \right)}{\partial \tau }|}_{\tau =\tau_{i}}=\frac{T_{Ni,i}\left(x,y\right)-T_{Ni,i-1}\left(x,y\right)}{d\tau }$$

(41) $${\frac{\partial T_{St}\left(x,y,\tau \right)}{\partial \tau }|}_{\tau =\tau_{i}}=\frac{T_{St,i}\left(x,y\right)-T_{St,i-1}\left(x,y\right)}{d\tau }$$

(42) $${\frac{\partial T_{w}\left(x,y,\tau \right)}{\partial \tau }|}_{\tau =\tau_{i}}=\frac{T_{w,i}\left(x,y\right)-T_{w,i-1}\left(x,y\right)}{d\tau }\mathrm{for}\;i=1,\ldots ,\;n_{t}$$

where $T_{Ni,i}\left(x,y\right)=T_{Ni}\left(x,y,\tau_{i}\right)$, $T_{St,i}\left(x,y\right)=T_{St}\left(x,y,\tau_{i}\right)$, and $T_{w,i}\left(x,y\right)=T_{w}\left(x,y,\tau_{i}\right)$. For *i* = 1, the initial conditions (27)–(29) are defined as:

(43) $$T_{Ni,0}\left(x,y\right)=f_{Ni}\left(x,y\right)$$

(44) $$T_{Ni,0}\left(x,y\right)=f_{Ni}\left(x,y\right)$$

(45) $$T_{w,0}\left(x,y\right)=f_{w}\left(x,y\right)$$

Applying the FD, the boundary value problem (BVP) at each time step is determined. The obtained BVPs are described by non-linear equations and the boundary conditions (conditions (37) and (39) are non-linear). We propose the implementation of Picard iterations, and we iteratively rewrite the governing equations:

(46) $$\frac{\partial^{2}T_{Ni,i}^{\left(j\right)}}{\partial x^{2}}+\frac{\partial^{2}T_{Ni,i}^{\left(j\right)}}{\partial y^{2}}=F_{Ni}\left(x,y,T_{Ni,i}^{\left(j-1\right)},T_{Ni,i-1}^{}\right)$$

(47) $$\frac{\partial^{2}T_{St,i}^{\left(j\right)}}{\partial x^{2}}+\frac{\partial^{2}T_{St,i}^{\left(j\right)}}{\partial y^{2}}=F_{St}\left(x,y,T_{St,i}^{\left(j-1\right)},T_{St,i-1}^{}\right)$$

(48) $$\frac{\partial^{2}T_{w,i}^{\left(j\right)}}{\partial x^{2}}+\frac{\partial^{2}T_{w,i}^{\left(j\right)}}{\partial y^{2}}=F_{w}\left(x,y,T_{w,i}^{\left(j-1\right)},T_{w,i-1}^{}\right)$$

where $T_{Ni,i}^{\left(j\right)}\left(x,y\right)$, $T_{St,i}^{\left(j\right)}\left(x,y\right)$, and $T_{w,i}^{\left(j\right)}\left(x,y\right)$ denote the temperatures of the superalloy, steel alloys, and weld at the *i*-th time step and *j*-th iteration, and:

$$\begin{array}{l} F_{Ni}\left(x,y,T_{Ni,i}^{\left(j-1\right)},T_{Ni,i-1}^{}\right)=\frac{{\rho c}_{Ni}\left(T_{Ni,i}^{j-1}\right)}{\lambda_{Ni}\left(T_{Ni,i}^{\left(j-1\right)}\right)}\frac{T_{Ni,i}^{\left(j-1\right)}\left(x,y\right)-T_{Ni,i-1}\left(x,y\right)}{d\tau }\\ -\frac{1}{\lambda_{Ni}\left(T_{Ni,i}^{\left(j-1\right)}\right)}{\frac{\partial \lambda_{Ni}\left(T_{Ni}\right)}{\partial T_{Ni}}|}_{T_{Ni}=T_{Ni,i}^{\left(j-1\right)}}({\left(\frac{\partial T_{Ni,i}^{\left(j-1\right)}}{\partial x}\right)}^{2}+{\left(\frac{\partial T_{Ni,i}^{\left(j-1\right)}}{\partial y}\right)}^{2}), \end{array}\;\begin{array}{l} F_{St}\left(x,y,T_{St,i}^{\left(j-1\right)},T_{St,i-1}^{}\right)=\frac{{\rho c}_{St}\left(T_{St,i}^{j-1}\right)}{\lambda_{St}\left(T_{St,i}^{\left(j-1\right)}\right)}\frac{T_{St,i}^{\left(j-1\right)}\left(x,y\right)-T_{St,i-1}\left(x,y\right)}{d\tau }\\ -\frac{1}{\lambda_{St}\left(T_{St,i}^{\left(j-1\right)}\right)}{\frac{\partial \lambda_{St}\left(T_{St}\right)}{\partial T_{St}}|}_{T_{St}=T_{St,i}^{\left(j-1\right)}}({\left(\frac{\partial T_{St,i}^{\left(j-1\right)}}{\partial x}\right)}^{2}+{\left(\frac{\partial T_{St,i}^{\left(j-1\right)}}{\partial y}\right)}^{2}), \end{array}\;\begin{array}{l} F_{w}\left(x,y,T_{w,i}^{\left(j-1\right)},T_{w,i-1}^{}\right)=\frac{{\rho c}_{w}\left(T_{w,i}^{j-1}\right)}{\lambda_{w}\left(T_{w,i}^{\left(j-1\right)}\right)}\frac{T_{w,i}^{\left(j-1\right)}\left(x,y\right)-T_{w,i-1}\left(x,y\right)}{d\tau }\\ -\frac{1}{\lambda_{w}\left(T_{w,i}^{\left(j-1\right)}\right)}{\frac{\partial \lambda_{w}\left(T_{w}\right)}{\partial T_{w}}|}_{T_{wl}=T_{w,i}^{\left(j-1\right)}}\left({\left(\frac{\partial T_{w,i}^{\left(j-1\right)}}{\partial x}\right)}^{2}+{\left(\frac{\partial T_{w,i}^{\left(j-1\right)}}{\partial y}\right)}^{2}\right) \end{array}$$

The boundary conditions (30)–(39) are still valid and are considered in the iteration process. Therefore, for each time step and iteration, the BVP i given by Equations (46)–(48) and (30)–(39) must be solved. 

The proposal of the paper is to apply one of the meshless methods, i.e., the method of fundamental solutions supported by approximation by the radial basis functions (RBF) and monomials.

It is assumed that the solutions of the BVP described by the inhomogeneous Equations (46)–(48)and the boundary conditions (30)–(39) are the sums of both the particular and homogeneous solutions:

(49) $$T_{Ni,i}^{\left(j\right)}=T_{p,Ni,i}^{\left(j\right)}+T_{h,Ni,i}^{\left(j\right)}$$

(50) $$T_{St,i}^{\left(j\right)}=T_{p,St,i}^{\left(j\right)}+T_{h,St,i}^{\left(j\right)}$$

(51) $$T_{w,i}^{\left(j\right)}=T_{p,w,i}^{\left(j\right)}+T_{h,w,i}^{\left(j\right)}$$

where $T_{p,Ni,i}^{\left(j\right)}$, $T_{p,St,i}^{\left(j\right)}$, and $T_{p,w,i}^{\left(j\right)}$ are the particular solutions, and $T_{h,Ni,i}^{\left(j\right)}$, $T_{h,St,i}^{\left(j\right)}$, and $T_{h,w,i}^{\left(j\right)}$ are the homogeneous solutions of the superalloy, steel, and weld, respectively, at the *i*-th time step and *j*-th iteration.

**Figure 7:**
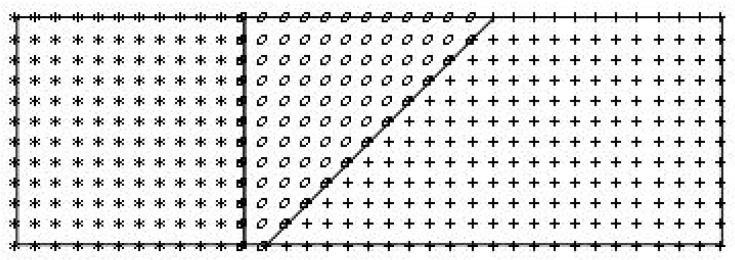
The sets of approximation points: the stars-the superalloy, the ovals–the weld, the point-crosses–the steel.

The particular solutions are obtained using approximation by the RBFs and monomials. The right-hand side of Equations (46)–(48) (inhomogeneous part) is approximated as follows:

(52) $$F_{Ni}\left(x,y,T_{Ni,i}^{\left(j-1\right)},T_{Ni,i-1}^{}\right)=\sum_{k=1}^{Na}a_{Ni,k}\phi \left(r_{Ni,k}^{\left(a\right)}\left(x,y\right)\right)+\sum_{k=1}^{Nm}a_{Ni,Na+k}p_{k}\left(x,y\right)$$

(53) $$F_{St}\left(x,y,T_{St,i}^{\left(j-1\right)},T_{St,i-1}^{}\right)=\sum_{k=1}^{Na}a_{St,k}\phi \left(r_{St,k}^{\left(a\right)}\left(x,y\right)\right)+\sum_{k=1}^{Nm}a_{St,Na+k}p_{k}\left(x,y\right)$$

(54) $$F_{w}\left(x,y,T_{w,i}^{\left(j-1\right)},T_{w,i-1}^{}\right)=\sum_{k=1}^{Na}a_{wl,k}\phi \left(r_{w,k}^{\left(a\right)}\left(x,y\right)\right)+\sum_{k=1}^{Nm}a_{w,Na+k}p_{k}\left(x,y\right)$$

where $\phi \left(r_{Ni,k}^{\left(a\right)}\left(x,y\right)\right)$, $\phi \left(r_{St,k}^{\left(a\right)}\left(x,y\right)\right)$, $\phi \left(r_{w,k}^{\left(a\right)}\left(x,y\right)\right)$ (for *k* = 1, …, *Na*) are the RBFs defined for the superalloy steel alloy and the weld, respectively, $p_{k}\left(x,y\right)$ (for *k* = 1, …, *Nm*) are monomials, $a_{Ni,k}$, $a_{St,k}$, and $a_{w,k}$ (for *k* = 1, …, *Na + Nm*) are real numbers, *Na* is the number of approximation points in each area $\Omega_{Ni}$, $\Omega_{St}$, and $\Omega_{w}$, and *Nm* is the number of monomials. The set of approximation points is defined in each area: $\left\{x_{Ni,k}^{\left(a\right)},y_{Ni,k}^{\left(a\right)}\right\}\subset \Omega_{Ni}$, $\left\{x_{St,k}^{\left(a\right)},y_{St,k}^{\left(a\right)}\right\}\subset \Omega_{St}$,$\left\{x_{w,k}^{\left(a\right)},y_{w,k}^{\left(a\right)}\right\}\subset \Omega_{w}$(for *k* = 1, …, *Na*), as presented in Figure 7. The points marked by stars are the approximation points of the superalloy, those marked by ovals are in the domain of the weld, and the point-crosses lie in the region of the steel zone. The quantities $r_{Ni,k}^{\left(a\right)}\left(x,y\right)$, $r_{St,k}^{\left(a\right)}\left(x,y\right)$, and $r_{w,k}^{\left(a\right)}\left(x,y\right)$ are defined as follows:

$$r_{Ni,k}^{\left(a\right)}\left(x,y\right)=\sqrt{{\left(x-x_{Ni,k}^{\left(a\right)}\right)}^{2}+{\left(y-y_{NI,k}^{\left(a\right)}\right)}^{2}}\;r_{St,k}^{\left(a\right)}\left(x,y\right)=\sqrt{{\left(x-x_{St,k}^{\left(a\right)}\right)}^{2}+{\left(y-y_{St,k}^{\left(a\right)}\right)}^{2}}\;r_{w,k}^{\left(a\right)}\left(x,y\right)=\sqrt{{\left(x-x_{w,k}^{\left(a\right)}\right)}^{2}+{\left(y-y_{w,k}^{\left(a\right)}\right)}^{2}}$$

The system of the linear algebraic Equations (52)–(54) is written for each approximation point (the proper domain) and solved. The solutions are the coefficients $a_{Ni,k}$, $a_{St,k}$, and $a_{w,k}$ (for *k* = 1, …, *Na + Nm*). Thus, the particular solutions are in the form of:

(55) $$T_{p,Ni,i}^{\left(j\right)}=\sum_{k=1}^{Na}a_{Ni,k}\psi \left(r_{Ni,k}^{\left(a\right)}\left(x,y\right)\right)+\sum_{k=1}^{Nm}a_{Ni,Na+k}P_{k}\left(x,y\right)$$

(56) $$T_{p,St,i}^{\left(j\right)}=\sum_{k=1}^{Na}a_{St,k}\psi \left(r_{St,k}^{\left(a\right)}\left(x,y\right)\right)+\sum_{k=1}^{Nm}a_{St,Na+k}P_{k}\left(x,y\right)$$

(57) $$T_{p,w,i}^{\left(j\right)}=\sum_{k=1}^{Na}a_{wl,k}\psi \left(r_{w,k}^{\left(a\right)}\left(x,y\right)\right)+\sum_{k=1}^{Nm}a_{w,Na+k}P_{k}\left(x,y\right)$$

where ψ(r) and $P_{k}\left(x,y\right)$ (for *k* = 1, …, *Nm*) are the particular solutions of Poisson’s equation with the inhomogeneous part of *ϕ*(*r*) and $p_{k}\left(x,y\right)$ (for *k* = 1, …, *Nm*), respectively.

The next step of the proposed procedure is the calculation of the homogeneous solution, which is obtained by applying the MFS. It is assumed that the homogeneous solutions are in the form of:

(58) $$T_{h,Ni,i}^{\left(j\right)}=\sum_{k=1}^{Ns}c_{Ni,k}fs\left(r_{Ni,k}^{\left(s\right)}\left(x,y\right)\right)$$

(59) $$T_{h,St,i}^{\left(j\right)}=\sum_{k=1}^{Ns}c_{St,k}fs\left(r_{St,k}^{\left(s\right)}\left(x,y\right)\right)$$

(60) $$T_{h,w,i}^{\left(j\right)}=\sum_{k=1}^{Ns}c_{wl,k}fs\left(r_{w,k}^{\left(s\right)}\left(x,y\right)\right)$$

where fs(r) is the fundamental solution function, $\left\{x_{Ni,k}^{\left(s\right)},y_{Ni,k}^{\left(s\right)}\right\}$, $\left\{x_{St,k}^{\left(s\right)},y_{St,k}^{\left(s\right)}\right\}$, and $\left\{x_{w,k}^{\left(s\right)},y_{w,k}^{\left(s\right)}\right\}$ are the sets of source points placed outside the regions $\Omega_{Ni}$, $\Omega_{St}$, and $\Omega_{w}$, respectively, and $r_{Ni,k}^{\left(s\right)}\left(x,y\right)=\sqrt{{\left(x-x_{Ni,k}^{\left(s\right)}\right)}^{2}+{\left(y-y_{Ni,k}^{\left(s\right)}\right)}^{2}}$, $r_{St,k}^{\left(s\right)}\left(x,y\right)=\sqrt{{\left(x-x_{St,k}^{\left(s\right)}\right)}^{2}+{\left(y-y_{St,k}^{\left(s\right)}\right)}^{2}}$, and $r_{w,k}^{\left(s\right)}\left(x,y\right)=\sqrt{{\left(x-x_{w,k}^{\left(s\right)}\right)}^{2}+{\left(y-y_{w,k}^{\left(s\right)}\right)}^{2}}$. *Ns* is the number of source points for each area, *s* is the distance between the boundaries $\Gamma_{Ni}$, $\Gamma_{St}$, and $\Gamma_{w}$, and the fictitious boundaries with the source points $c_{Ni,k}$, $c_{St,k}$, and $c_{w,k}$ (for *k* = 1, …, *Ns*) are real numbers.

The forms of solutions (49)–(51) are introduced to the boundary conditions (30)–(39), and the approximated form of the homogeneous solutions (58)–(60) is included. Thus, the boundaries $\Gamma_{Ni}$, $\Gamma_{St}$, and $\Gamma_{w}$ are discretized, i.e., the sets of boundary points are defined as: $\left\{x_{Ni,k}^{\left(b\right)},y_{Ni,k}^{\left(b\right)}\right\}\subset \Gamma_{Ni}$, $\left\{x_{St,k}^{\left(b\right)},y_{St,k}^{\left(b\right)}\right\}\subset \Gamma_{St}$ and $\left\{x_{w,k}^{\left(b\right)},y_{w,k}^{\left(b\right)}\right\}\subset \Gamma_{w}$ (for *k* = 1, …, *Nb*), where *Nb* is the number of boundary points at each boundary. Additionally, for each boundary point, the proper boundary condition is written. This gives the system of linear algebraic equations with the unknowns $c_{Ni,k}$, $c_{St,k}$, and $c_{w,k}$ (for *k* = 1, …, *Ns*). The solution of this equations system completes the procedure of finding the solution in the *i*-th time step and *j*-th iteration.

**Figure 8:**
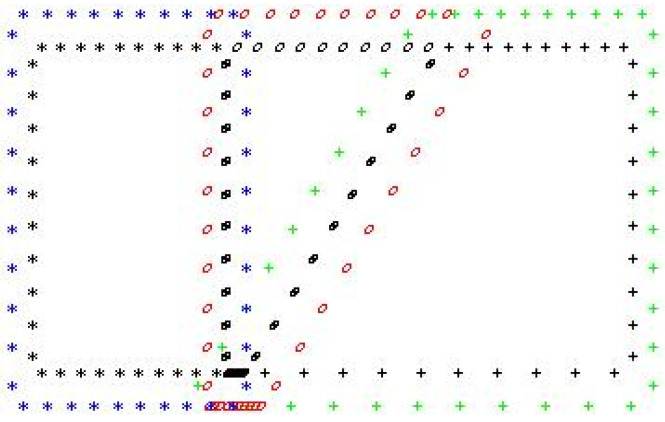
The sets of boundary and source points: the black points–the boundary points, black stars–the boundary region for superalloy, the black ovals–the weld boundary, the black crosses–steel boundary zone, the blue stars-the fictitious contour of the superalloy, the red ovals–the fictitious shape of the weld, the green crosses–the fictitious contour of the steel.

The idea of the selection of boundary and source points is presented in Figure 8. The black points are the boundary points. The black stars denote the boundary of the superalloy, the black ovals lie on the weld boundary, and the black crosses denote the boundary steel zone. The source points are also plotted in Figure 8. The blue stars denote the fictitious contour of the superalloy, the red ovals lie on the fictitious weld shape, and the green crosses denote the fictitious contour of the steel zone. The distance between the boundary and fictitious contour *s* is clearly indicated. Therefore, for this case the following sentence can be formulated: different patterns of the points reflects sections of single bevel joint.

The procedure of the iterations at the *i*-th time step is stopped if the following condition is fulfilled:

(61) $$\max\left\{\varepsilon_{Ni},\varepsilon_{St},\varepsilon_{w}\right\}<\varepsilon$$

where

(62) $$\varepsilon_{Ni}=\frac{1}{Nt}\sum_{k=1}^{Nt}\left|T_{Ni,i}^{\left(j\right)}\left(x_{Ni,k}^{\left(t\right)}-y_{Ni,k}^{\left(t\right)}\right)-T_{Ni,i}^{\left(j-1\right)}\left(x_{Ni,k}^{\left(t\right)}-y_{Ni,k}^{\left(t\right)}\right)\right|$$

(63) $$\varepsilon_{St}=\frac{1}{Nt}\sum_{k=1}^{Nt}\left|T_{St,i}^{\left(j\right)}\left(x_{St,k}^{\left(t\right)}-y_{St,k}^{\left(t\right)}\right)-T_{St,i}^{\left(j-1\right)}\left(x_{St,k}^{\left(t\right)}-y_{St,k}^{\left(t\right)}\right)\right|$$

(64) $$\varepsilon_{w}=\frac{1}{Nt}\sum_{k=1}^{Nt}\left|T_{w,i}^{\left(j\right)}\left(x_{w,k}^{\left(t\right)}-y_{w,k}^{\left(t\right)}\right)-T_{w,i}^{\left(j-1\right)}\left(x_{w,k}^{\left(t\right)}-y_{w,k}^{\left(t\right)}\right)\right|$$

where $\left\{x_{Ni,k}^{\left(t\right)},y_{Ni,k}^{\left(t\right)}\right\}\subset \Omega_{Ni}\cup \Gamma_{Ni}$, $\left\{x_{St,k}^{\left(t\right)},y_{St,k}^{\left(t\right)}\right\}\subset \Omega_{St}\cup \Gamma_{St}$, and $\left\{x_{w,k}^{\left(t\right)},y_{w,k}^{\left(t\right)}\right\}\subset \Omega_{w}\cup \Gamma_{w}$ (for *k* = 1, …, *Nt*) are the sets of trial points placed in each area, and on its boundary, *Nt* is the number of trial points in each area and boundary, and ε is a small number.

### 3.3. Validation of the Numerical Procedure and Numerical Results

Computer simulations were carried out using a computer program written by the authors of the article. The numerical calculation stage was performed to validate the proposed numerical approach. The proper values of the numerical procedure parameters were chosen to obtain the demanded results. These results of the numerical simulations were compared with the real data. Then, the different values of the parameters of the cooling procedure were tested. The aim was to obtain as a heat-affected zone that was as small as possible.

The dimensions of the specimens were expressed as the following values: *h* = 8 mm, *d_Ni_* = 30 mm, *d_w_* = 2 mm, and *d_St_* = 30 mm.

The cooling process was performed using a micro-jet system. The values of the welding velocity and the cooling process were equal to 240 mm/min. The gas used was helium. The other parameters of the cooling system were the cooling jet velocity *v* (related to the pressure of the gas) and the diameter of the micro-jet injector *d_j_*. Thus, the four variants of the cooling system were tested.

These variants were:

Variant A: *p* = 0.4 MPa, *d_j_*. = 50 μm;

Variant B: *p* = 0.4 MPa, *d_j_*. = 60 μm;

Variant C: *p* = 0.5 MPa, *d_j_*. = 50 μm;

Variant D: *p* = 0.5 MPa, *d_j_*. = 60 μm.

For the application of the MFS, the special function, called the fundamental solution, for the differential operator in the governing equations has to be known. The same operator, the Laplace one, is applied in all the governing Equations (46)–(48). The fundamental solution for the Laplace operator is given by the formula:

*fs*(*r*) = ln(*r*), (65)

where *r* is the distance from one of the source points and the chosen point that lies in the domain in which the equations are fulfilled. 

To apply the approximation given by Equations (52)–(54), the radial basis functions must be chosen. In this numerical implementation, the thin-plate spline RBF is chosen, and seven monomials are used. The thin-plate spline is expressed as:

*ϕ*(*r*) = *r*^2^ ln(*r*) (66)

The parameters of the numerical method were selected. The selection was based on some simulations using the trial values of the method parameters (number of boundary points, number of source points, distance between the real and source contours). This calibration procedure was performed for the cooling variant D. Ten time steps were calculated, and at each time step, some iterations were performed. The number of iterations depends on the criterion’s fulfillment (61).

The validation procedure consists of simulations using different values of the method parameters and checking the mean square error applied to the boundary points. The fulfillment of the boundary conditions by the obtained solution was tested for every iteration at each time step. As an example of the values, the mean square error for the 5th time step and 3rd iteration is presented in Table 3. For every calculated time step and iteration, the error had the lowest value for the number of boundary points equal to 40, and the distance between the boundary and fictitious boundary was equal to 0.2. The parameter values were selected if the mean square error had the smallest value.

The trial calculations were also performed to select the number of approximation points in the regions (superalloy, steel, and weld). This selection is conducted by calculating a mean square error of the fulfillment of the right-hand-side functions of Equations (46)–(48) by approximations (52)–(54). The values of the mean square error of the approximation error for the 5th time step and 3rd iteration are presented in Table 4. As we can observe, the lowest value appears for the number of approximation points equal to 121. The other values of the mean square error calculated for the rest of the time steps and iterations were lowest when 121 approximation points were defined. 

Finally, the following parameters were chosen: the number of boundary points was 40 (in each considered region); the number of source points was 121 (in each considered area); and the distance between the real and fictitious boundary was 0.2 (in each considered area). 

For this referential example, the values of (61)–(64) were also calculated for the set of trial points. Thus, it was observed that values of (61)–(64) of less than 10^−5^ were achieved for a relatively low number of iterations (see Table 5). The number of calculated iterations is presented in Table 6, which was prepared for the first two time steps. As concluded in Table 6, the proposed numerical procedure requires little work.

After determining the parameters of the numerical procedure, the numerical experiment can be started. The range of the heat-affected zone of the superalloy and steel alloys was simulated. The calculations were made for the welding parameters given in the natural experiment. 

**Figure 9:**
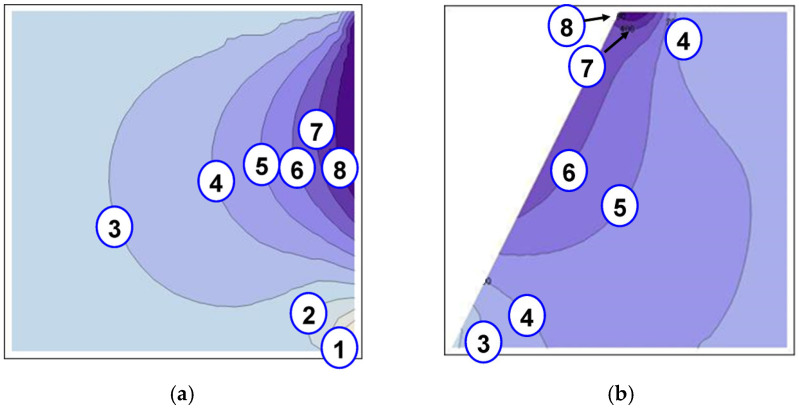
(**a**). Temperature distribution at the time step of maximum cooling: (**a**) the superalloy, (**b**) the steel alloy, 1—1000 °C, 2—900 °C, 3—800 °C, 4—700 °C, 5—600 °C, 6—500 °C, 7—400 °C, and 8—300 °C.

Thus, the temperature distribution during the welding and cooling was calculated for all the variants of the cooling process. The distribution of the temperature with the cooling variant D is presented in Figure 9a for the superalloy and Figure 9b for the steel alloy. The graphs were created for the time step in which the maximum cooling appeared.

**Figure 10:**
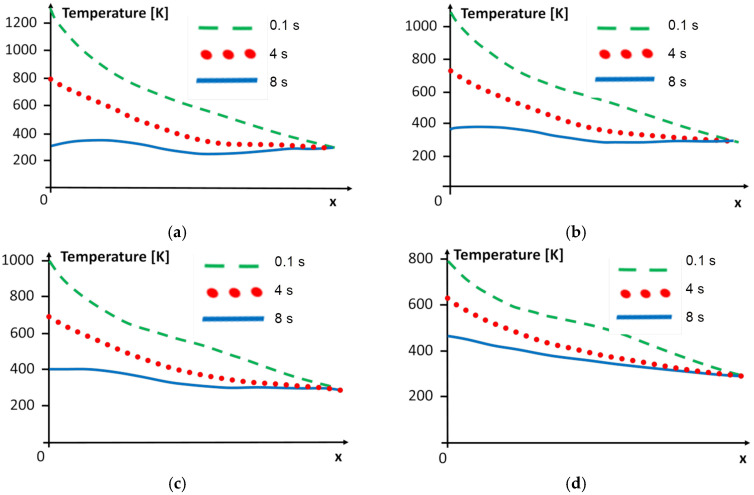
Temperature distribution in the steel along axis *x* for y = 0.5 mm for (**a**) variant A, (**b**) variant B, (**c**) variant C, and (**d**) variant D of the cooling system.

**Figure 11:**
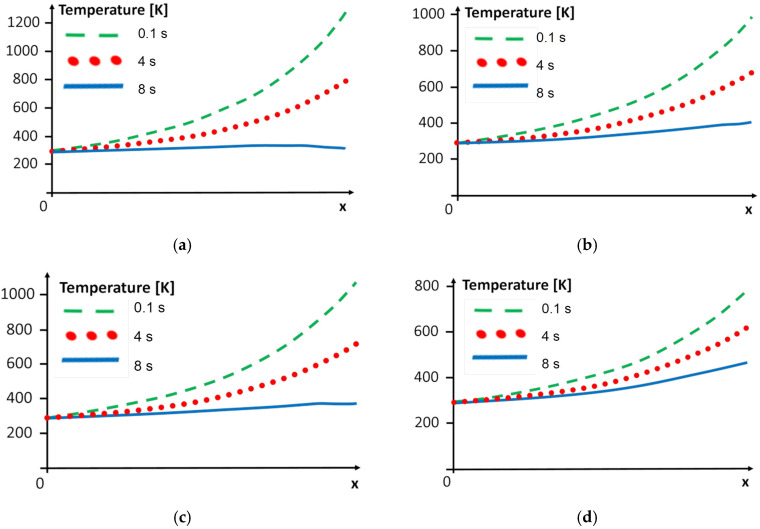
Temperature distribution in the superalloy along axis *x* for y = 0.5 mm for (**a**) variant A, (**b**) variant B, (**c**) variant C, and (**d**) variant D of the cooling system.

We can also observe changes in the temperature over time. The graphs presented in Figure 10 show the temperature at the chosen time steps, obtained by applying different variants of the cooling system to the steel region. The graphs (Figure 11) show the temperature at the selected time steps obtained using another variant of the cooling system in the superalloy region.

In every graph in Figure 10 and Figure 11, we can observe that the boundary condition at the free end is fulfilled, i.e., the temperature should be equal to the ambient temperature for *x* = 0 in the superalloy and for *x* = *d_Ni_* + *d_w_* + *d_St_* in the steel zone. At the other ends of both areas, the temperature is dependent on the cooling of the weld area, and it is also straightforward to observe that these boundary conditions are fulfilled.

From the engineering point of view, it is interesting to estimate the heat-affected zone of welded materials. The process of cooling and decreasing the HAZ has an important role, and the rate of cooling determines the welded materials’ characteristics. Observing Figure 10 and Figure 11, we found changes in the range of the HAZ in the superalloy and steel zones. The temperature limit of 773 K was considered to examine the range of the heat influence zone. The content of the HAZ for the chosen time steps is presented in Table 7.

The numbers presented in Table 7 are the length of the zone with a temperature higher than 773 K divided by the vertical dimension of the specimens. We can observe that the HAZ decreases fastest with cooling variant D, and the slightest decrease in the HAZ appeared when cooling variant A was applied. Additionally, variants B and C give similar decreasing rates of the HAZ, while variant B yields a smoother cooling.

**Figure 12:**
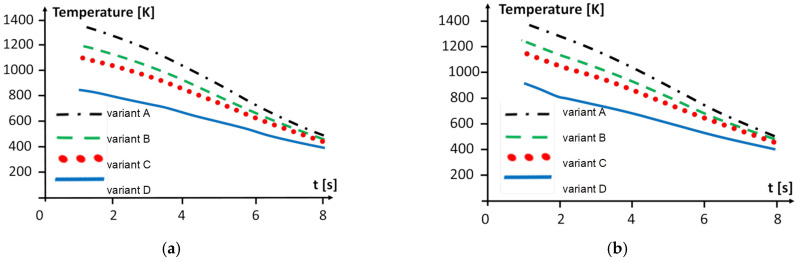
Temperature distribution at points in the HAZ (**a**) in the superalloy region and (**b**) in the steel region.

It is most interesting to simulate and compare the processes for all the four variants of the cooling after welding. The simulation results are presented in Figure 12a for the superalloy, Figure 12b for the steel alloy). It shows the temperature distribution at specific points (the points are placed in the HAZ, 0.5 mm from the welded and cooled boundary of each welded material, i.e., the superalloy and steel alloys).

We can observe that the most intensive cooling is obtained by variant D for both metal regions, but the heating and cooling progress faster in the steel alloy compared to the superalloy region. 

The parameter known in the literature which demonstrates the efficiency of the cooling system is the cooling rate. The computer simulations make it possible to obtain the precise weld cooling conditions of the welding process, especially in the range of 500–800 °C. The cooling rate parameter is presented in Table 8. One can observe the cooling rate according to the four variants of the nozzle diameter and gas pressure in each welded material: the steel and superalloy. The highest values are those for the cooling by the D variant. Two of them, B and C, give similar results. The slowest cooling appears for variant A. However, the cooling process should not be too intensive. It is crucial to note that the cooling rate of the steel region is lower than that of the superalloy zone. This means that the cooling process is more intensive in the superalloy region. Therefore, the parameters of the micro-jet cooling process should be selected based on the steel region in order to obtain the intensity of the cooling, which gives the demanded HAZ. 

### 3.4. Comparison with Another Numerical Approach

In engineering simulations, the finite element method (FEM) is often used. The considered problem is given by Equations (7)–(22), which were solved using the software based on the FEM. Computer simulations based on the FEM were carried out using a computer program written by the authors of the article. It was effortless to introduce the geometry of the specimens. It required some effort to define the temperature-dependent material characteristics and proper initial boundary conditions and coefficients in the governing equations. Using FEM software computations, the domains of the materials were divided into a large number of elements. Triangular elements with three nodes were used in the calculations. Each domain was divided into 10,000 elements. This caused the system of algebraic equations to be extensive (the number of equations was proportional to the number of 3 × 10,000 nodes in the base functions, i.e., at least 90,000 equations) compared to what we observed in the case of the MFS. Using the proposed algorithm, two systems of algebraic equations were solved. One method of linear algebraic Equations (52)–(54) was used for the calculation of the particular solutions. The number of equations was3 × (121 + 7) = 384. The second set of equations were used for the calculation of the homogeneous solutions (58)–(60), which consisted of 100 equations. Due to such a low number of equations in the system, the proposed algorithm based on MFS is less time-consuming than the calculations performed using FEM. MFS is a meshless method, and RBF does not require the construction of any mesh in the considered region. Only a cloud of points is defined in the considered areas, and only the boundaries of the areas are subject to discretization (the dimension of the problem is reduced by 1). However, in FEM, the procedure of creating the mesh, defining the element, and the basis functions must be applied. This procedure is also highly time-consuming. Moreover, the MFS approach requires less memory (for the data collection and for the processor to perform calculations) than FEM. The values of the other parameters of the procedure based on FEM, analogous to the parameters of the proposed procedure based on MFS, were assumed to be the same in both simulations. The parameters, having been adopted in this way, allowed us to obtain numerically stable and convergent procedures. Both methods deliver results in the form of continuous analytical functions. This is a very useful property, because it allows for the performance of post-processing analysis. The possibility of such an analysis is significant from an engineering point of view. The advantage of the MFS approach is that the analytical functions, which construct the solutions, have derivatives of the order of infinity. In FEM, this aspect depends on the used basis functions, but in the general approach, the basis functions possess derivatives of a few first orders.

A comparison of the results obtained using both procedures, the MFS-based and FEM-based methods, is presented in Table 9. The calculations were performed for variant C of the cooling process. The values of the temperature calculated at the point of half of the specimen height and 0.5 cm from the edge of the welded edges (for the superalloy and steel zones) are provided. As can be observed, the differences between the calculated values using the different numerical procedures are not substantial. However, the comparison of the time and memory consumption using both procedures has to be taken into account as well. In our computations, the algorithm based on MFS is more appropriate. It gives the same results as FEM but is less time- and memory-consuming.

The other method used to solve initial boundary engineering problems is the finite differences method (FDM). This method partially supported the algorithm proposed in this paper. The basic form of the FDM is dedicated to linear problems. For the purpose of using the FDM to solve all the aspects of the considered problems of welding and cooling, the other numerical methods should be implemented as well. However, there is a fundamental imperfection of the FDM. This method gives results in a discrete form and not a continuous analytical function. Thus, additional procedures have to be applied to perform the engineering post-processing analysis. For this purpose, the interpolation technique is implemented. However, such an approach does not ensure the compatibility of the used interpolation function with the character of the calculated phenomenon. Due to this shortcoming of the FDM, the calculations performed by this method were omitted.

### 3.5. The Application of the MFS-Based Procedure

In the previous section, it was validated that the proposed MFS-based numerical algorithm is a good tool for simulating the time-dependent temperature field in welded materials. Thus, the method may be applied to estimate the cooling process parameters so as to obtain workpieces with the demanded characteristics (according to the analysis of the HAZ or cooling rate). It is also possible to simulate the time-dependent welding–cooling process for other materials. The procedure was elaborated for the welding of two different materials with non-linear characteristics dependent on the temperature. 

The simulation test was performed using materials with thermal conductivity coefficients and a specific heat capacity different from those of the materials defined in the validation section.

Thus, it is assumed that the superalloy is replaced by materials with a thermal conductivity coefficient and specific heat capacity equal to the characteristics of the alloy, multiplied by 0.8, 0.9, 1.1, and 1.2. This means that:

*λ*_I,1_ = 0.8 *λ_Ni_*, *λ*_I,2_ = 0.9 *λ_Ni_*, *λ*_I,3_ = 1.1 *λ_Ni_*, *λ*_I,4_ = 1.2 *λ_Ni_*,

where the first subscript, which is equal to I, refers to the material replacing the superalloy, and the second one describes the version of the material. 

The parallel change is performed for the steel, replaced by the material with notation II. Additionally, the characteristics of the variant of material II are noted as:

*λ*_II,1_ = 0.8 *λ_St_*, *λ*_II,2_ = 0.9 *λ_St_*, *λ*_II,3_ = 1.1 *λ_St_*, *λ*_II,4_ = 1.2 *λ_St_*,

The parameters of the applied numerical procedure (including the parameters related to the welding–cooling process) are not changed. They stay the same as in the validation procedure. 

For such modified materials, the values of the cooling rate parameter are calculated, and they are included in Table 10.

The cooling rate parameter has values in the range of 50–80 (which is most popularly demanded by engineers) for cooling variants B and C. The materials’ characteristics are changed slightly compared to the input ones (given by Formulas (1), (2), (4), and (5)). Thus, we can also conclude that cooling schemes B and C are the most advantageous. More concerning is the fact that the cooling rate of variant D becomes very high (higher than 180). This may indicate that cracks could appear in the welded material. In Section 3.3, a high cooling rate appeared, and in chapter 2, it was pointed out that there were no cracks. However, in the case of this section, regarding the cooling, the rate is much higher, and there is no reliable assurance that imperfections (such as cracks) will not appear.

## 4. Validation In Situ and Discussion

To validate the results obtained by the numerical simulations, some mechanical tests were performed.

Once the joints were welded with various micro-jet cooling parameters, non-destructive tests were carried out, including visual, penetration, and ultrasonic tests. 

**Figure 13:**
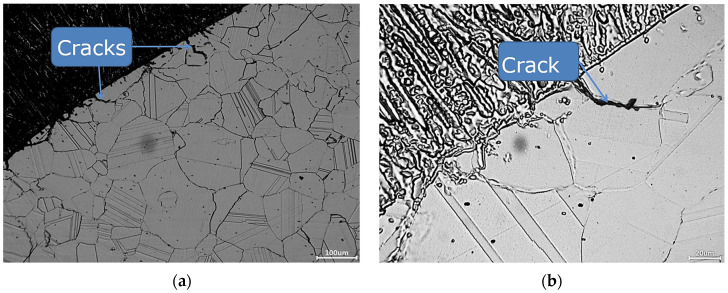
Microstructure of the specimens obtained by welding without the micro-jet cooling process. (**a**) the microstructure in a general view; (**b**) the microstructure in a view on the crack.

The following results were collected:

- Minor cracks appeared in the joints made using the MIG process, without micro-jet cooling (Figure 13a,b);
- No defects or non-conformities for level B (according to the PN-EN ISO 5817:2005 standard) appeared in the joints made using the MIG process, where helium micro-jet cooling was applied.

**Figure 14:**
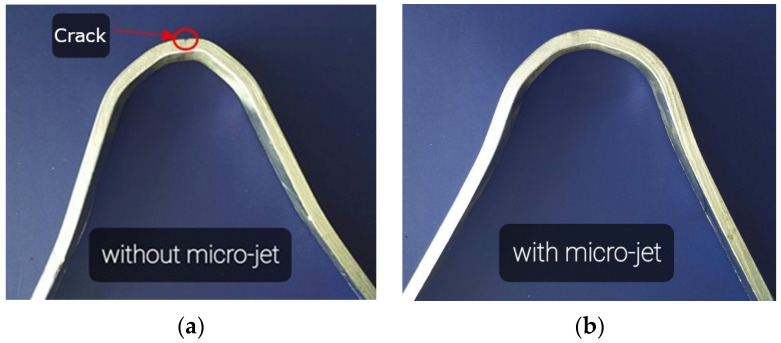
View of the welding specimens after the bending test: (**a**) the specimen made without micro-jet cooling, (**b**) the specimen made with micro-jet cooling.

The next step for the joint quality assessment was performed by applying destructive tests (bending, tensile, hardness, microscopic observations). In the case of all the specimens, the bending test was performed. Five bending tests were conducted from the face and the root side of the weld. In the joints made using the classic MIG process, cracks in the weld were observed at a bending angle above 140° (Figure 14a).

In the case of the application of micro-jet cooling in the MIG process of connecting Alloy 59 with the S355J2 hard-rusting steel, after the welding, no cracks or other incompatibilities were detected in the tested specimens (Figure 14b) The bending test results showed that the joints were welded correctly, reaching the required quality.

**Figure 15:**
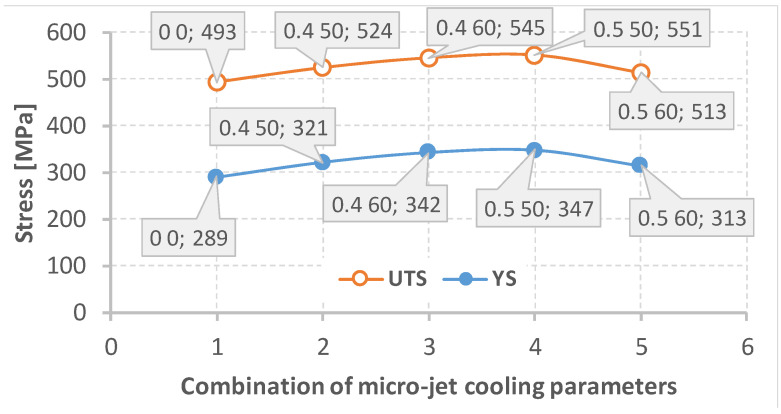
Variations in the ultimate tensile strength (UTS) and yield stress (YS) due to the parameters of the micro-jet cooling based on the data presented in Table 11.

Next, tensile tests were carried out. The obtained results for the mixed joint made of Alloy 59 and hard-rusting S355J2 steel prepared using MIG and different micro-jet cooling parameters (average from three measurements) are presented in Table 11, Figure 15.

The data analysis (Table 11, Figure 15) shows that all the joints made with the use of cooling had comparable mechanical properties (a yield stress above the desired value of 300 MPa and ultimate tensile strength above 500 MPa). These data follow the mechanical parameters of the S355J2W defined by the material manufacturers [39]. In the connections for which micro-jet cooling was not introduced, worse mechanical properties were noted. These could have been influenced by micro-cracks that did not exist in the joints made using micro-jet cooling. The best mechanical properties of the mixed (Alloy 59 with S355J2 steel) joint were obtained using the following micro-jet cooling parameters: for the helium cooling gas, a gas pressure of 0.5 MPa and a stream diameter of 50 µm.

**Figure 16:**
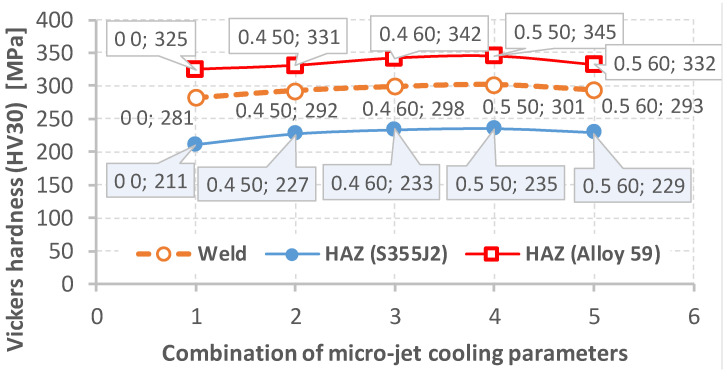
Variations in the Vickers hardness (HV30) as an effect of the parameters of the micro-jet cooling based on the data presented in Table 12.

**Figure 17:**
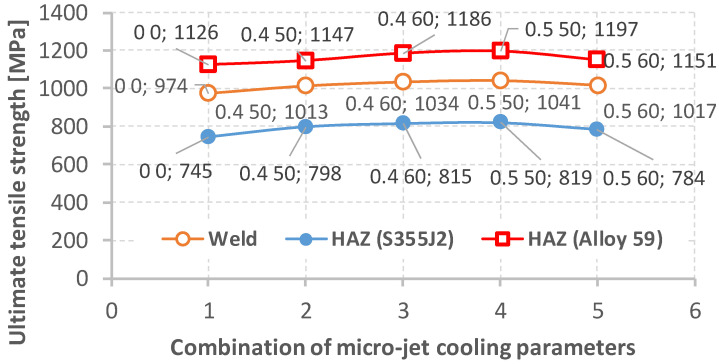
Variations in the ultimate tensile strength as an effect of the parameters of the micro-jet cooling based on the data presented in Table 12.

Hardness tests of the Alloy 59 joint with S355J2 hard-rusting steel were also used to extend our knowledge of the joint quality. The hardness of the steel was 185 MPa, while in the case of the superalloy, this parameter reached 375 MPa. Table 12 and Figure 16 show the results of the hardness tests of the heat-affected zone (HAZ) for both the welded sides and the weld hardness of the tested joints (using different micro-jet cooling parameters). Brinell hardness data calculated based on the Vickers hardness results for the HAZ were utilized to assess the values of the ultimate tensile strength for the three areas considered, as shown in Table 12, Figure 17. For this purpose, the factor equal to 3.53 as a proportion between the UTS and the hardness value was determined. In this case, the smallest value of the mechanical parameter was equal to 745 MPa for the joint region represented by the S355J2 steel. The highest value was obtained for the superalloy side, expressing 1197 MPa. Following these data, it can be determined that the hardness values become lower from the superalloy side and through the joint material up to the steel side. From a practical point of view, the steel and superalloy regions can be called the strongest and weakest ones, respectively. In regard to inspection, this means that the steel region is representative of the observation of cracks and deformation. 

The data presented in the table show that the hardness values of all the joints made using micro-jet cooling are comparable. The joints made without micro-jet cooling are characterized by the slightly lower hardness of the joint in both heat-affected zones.

**Figure 18:**
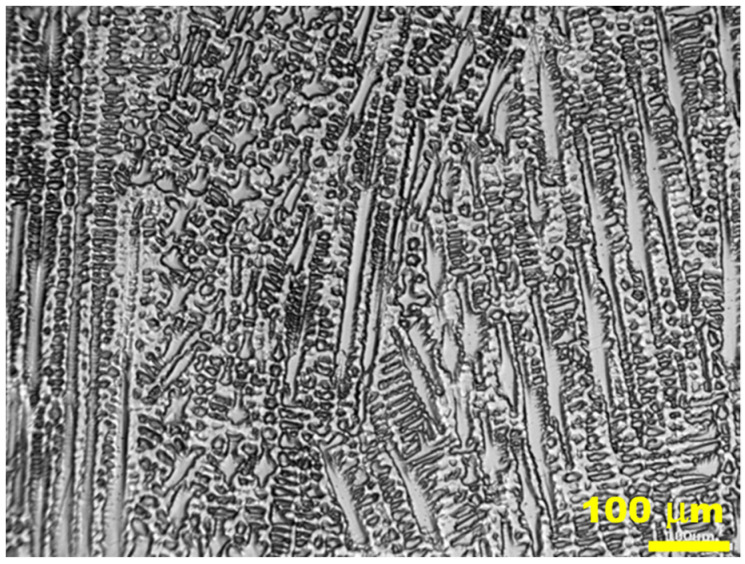
The microstructure of the middle part of the mixed joint (S355J2W steel with Alloy 59).

Figure 18 illustrates the microstructure of the mixed weld made using the MIG process with the application of micro-jet cooling.

**Figure 19:**
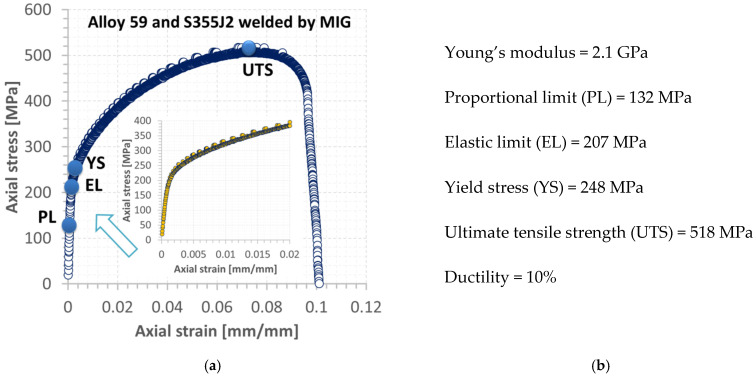
Tensile characteristics of the MIG mixed weld (**a**) and mechanical parameters (**b**).

**Figure 20:**
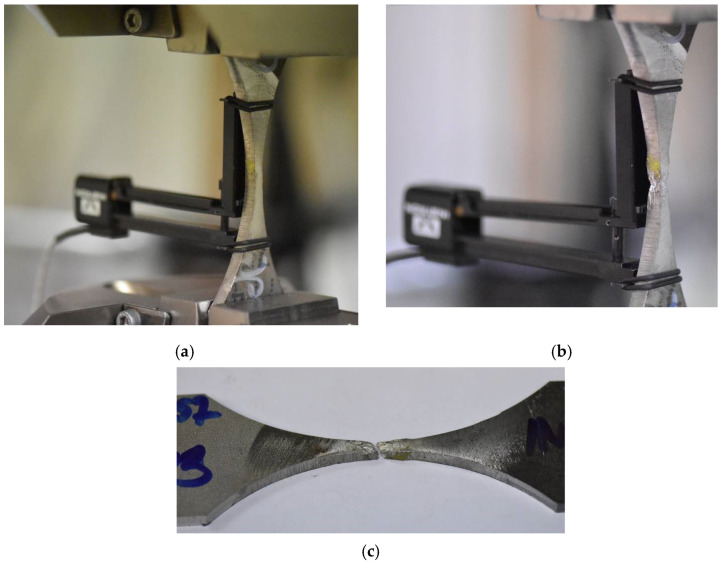
Specimen with the MIG mixed weld: (**a**) before and (**b**,**c**) after the tensile test, respectively.

The weld manufactured with the application of micro-jet cooling has an austenite dendritic cell microstructure. This guarantees better plastic properties, derived from the column microstructure. No cracks were observed in the junction with the dendritic cellular structure. The influence of the cooling rate on the structure of the welded material was considered in [40]. This is confirmed by the results obtained from the tensile test, observing the number of data collected upon fracture, the ductility value (Figure 19a,b, respectively), and the fracture zone (Figure 20b,c). 

These results directly demonstrate that the weld’s fracture under monotonic tension occurred upon no dynamic reaction of the material examined, compared to the structural steel. It can be concluded the MIG process, when applied to the Alloy 59 and S355J2W joint, is very beneficial, as there is no brittle cracking and no rapid development of cracks before the separation.

## 5. Summary and Conclusions

The welding of two types of structural materials (Alloy 59 and S355J2W structural steel) is a challenging task, but in an in situ experiment using a micro-jet cooling process, the problem can be solved. Here, the parameters of the welding and cooling process were discussed, and the results of the in situ experiments were presented and analyzed. The data and the experimental results are the basic steps for the numerical stage. Based on the conception of the numerical approach, the method for the calculation can be deemed as innovative. The proper combination of the FDM, Picard iterations, approximation by RBS, and MFS was implemented to solve the heat transfer problem during the welding–cooling process. The validation of the proposed numerical method was performed according to the results of the in situ experiments. The theoretical research, performed based on the proposed numerical algorithm, allows for the estimation of the characteristics of the resultant specimen. It is also possible to select welding–cooling process parameters to obtain workpieces with the required values of the mechanical properties. 

Analyzing the results collected in the non-destructive and destructive tests, the following conclusions can be formulated:

- Micro-jet cooling used in the MIG process enables one to obtain a correctly mixed joint, such as that of the S355J2 steel and Alloy 59.
- Excellent mechanical properties of the joint were achieved, i.e., a high yield stress (248 MPa) and ultimate tensile strength (518 MPa, ductility 10%).
- The inspection confirmed no welding defects or incompatibilities, such as sticking, fusion, cracks, or porosity.
- The in situ experiments showed that the best properties of the welded material can be obtained by welding with cooling variant C. The values of the cooling rate (60 for the superalloy and 71 for the steel zone) of variant C (presented in Table 9) are the most beneficial from the engineering point of view. It is known that, for such values of the cooling rate, the welding process gives the best-quality joints. There are no cracks, and the obtained materials have good mechanical and plastic properties. This was confirmed by our in situ experiments.
- The numerical algorithm based on the MFS is sufficient, because it requires less time and PC memory than the other methods (i.e., using meshing and the discrete approach).
- The method can also be used for other types of joints made of metallic structural materials, including hard-welded ones, such as high-strength steels and their advanced grades.
- The algorithm can be used in the welding calibration process when two different structural materials are taken for joining. It is also very suitable for training courses for welding engineers.

The applied calculation method is offers new possibilities for the use of equations based on a free approach and is closely related to the physics of the phenomena associated with the manufacturing of joints by welding using the micro-jet cooling technique as well. Observing the results of the numerical experiment, it is acceptable to state the proposed method based on the method of fundamental solutions is the proper tool for solving the problem of multi-material welding with micro-jet cooling.

The proposed method of joining steel-nickel alloy can be tested in situ and numerically, showing that this material can be used for the critical elements of power plant devices.

## Figures and Tables

**Table 1:** Materials used to build the rotor platforms: mechanical properties [6].

Material	Yield Stress (YS) (MPa)	Ultimate Tensile Strength (UTS) (MPa)	Elongation A_5_ (%)
Alloy 59	950	1150	40
Steel S355J2	355	570	5
Welding wire NiCr23Mo16	450	720	35

**Table 2:** Materials used to build the rotor platforms: chemical composition [6].

Material	C%	Si%	Mn%	Mo%	Nb	Cr%	Ni%	Fe%
2.4819	0.004	0.05	0.02	15	-	16.16	remains	6.07
S355J2	0.2	0.4	1.50	-	-	-	0.3	remains
Welding wire NiCr23Mo16	0.015	0.05	0.6	16	0.03	23	remains	0.5

**Table 3:** Mean square error for fulfilling the boundary conditions.

*Nb* = *Ns* *s*	20	40	60	80
0.1	4 × 10^−6^	2 × 10^−6^	5 × 10^−6^	6 × 10^−6^
0.2	6 × 10^−7^	1 × 10^−7^	5 × 10^−7^	7 × 10^−7^
0.3	4 × 10^−6^	3 × 10^−6^	4 × 10^−6^	6 × 10^−6^
0.4	2 × 10^−4^	1 × 10^−4^	3 × 10^−4^	7 × 10^−4^
0.5	5 × 10^−4^	3 × 10^−4^	7 × 10^−4^	9 × 10^−4^

**Table 4:** Mean square error for approximation by RBFs.

*Na*	25	49	121	144	169
Mean square error	4 × 10^−4^	3 × 10^−6^	4 × 10^−9^	5 × 10^−9^	9 × 10^−8^

**Table 5:** Values of $\varepsilon_{Ni}$, $\varepsilon_{St}$, and $\varepsilon_{w}$.

*i*-th Time Step	*j*-th Iteration	$\varepsilon_{Ni}$	$\varepsilon_{St}$	$\varepsilon_{w}$
1	2	4 × 10^−5^	5 × 10^−5^	5 × 10^−5^
	3	2 × 10^−5^	2 × 10^−5^	3 × 10^−5^
	4	2 × 10^−5^	1 × 10^−5^	2·× 10^−5^
	5	8 × 10^−6^	7 × 10^−6^	8 × 10^−6^
2	2	7 × 10^−5^	5 × 10^−5^	5 × 10^−5^
	3	5 × 10^−5^	4·× 10^−5^	3 × 10^−5^
	4	4 × 10^−5^	3 × 10^−5^	2 × 10^−5^
	5	2 × 10^−5^	2 × 10^−5^	1 × 10^−5^
	6	8 × 10^−6^	7 × 10^−6^	7 × 10^−6^

**Table 6:** Numbers of iteration steps.

Time Step	1	2	3	4	5
Number of iterations	5	6	6	6	7

**Table 7:** Range of the HAZ.

Material	Alloy 59		S355J2W Steel	
Time	0.1 s	4 s	0.1 s	4 s
Variant A	0.2182	0.0727	0.0909	0.0000
Variant B	0.2909	0.0182	0.2182	0.0182
Variant C	0.2545	0.0182	0.1818	0.0182
Variant D	0.3636	0.0545	0.3272	0.0545

**Table 8:** The cooling rate parameters of the simulated cooling.

Cooling Type	A	B	C	D
Steel region	47	58	60	150
Superalloy region	50	69	71	171

**Table 9:** Values of the temperature at the chosen point in the superalloy and steel zone.

Time (s)	Alloy 59			S355J2 Steel		
	MFS	FEM	Absolute Value of Difference	MFS	FEM	Absolute Value of Difference
**1**	1100.11	1099.80	0.31	1150.52	1150.64	0.12
**2**	1024.82	1025.15	0.33	1049.80	1049.71	0.09
**4**	850.25	850.29	0.04	850.05	850.10	0.05
**6**	624.79	624.81	0.02	649.70	649.64	0.06
**8**	474.89	475.01	0.12	450,10	450.05	0.05

**Table 10:** The cooling rate parameters for the modified materials.

Variant Version of Material		A	B	C	D
**Material I**	1	47	65	67	160
	2	49	67	69	166
	3	52	72	73	180
	4	55	75	77	189
**Material II**	1	43	54	55	135
	2	45	57	58	137
	3	50	61	62	152
	4	53	64	65	152

**Table 11:** Mechanical properties of the mixed joints.

Micro-Jet Gas Pressure, (MPa)	Micro-Jet Stream Diameter, (µm)	Yield Stress, (MPa)	Ultimate Tensile Strength, (MPa)
without	without	289	493
0.4	50	321	524
0.4	60	342	545
0.5	50	347	551
0.5	60	313	513

**Table 12:** Vickers (HV30), Brinell hardness test result and values of ultimate tensile strength (UTS).

Micro-Jet Gas Pressure (MPa)	Micro-Jet Stream Diameter (µm)		HAZ from the Steel Side S355J2 (MPa)		Weld (MPa)	HAZ from the Alloy 59 Side (MPa)	
without	without		211		281	325	
0.4	50		227		292	331	
0.4	60		233		298	342	
0.5	50		235		301	345	
0.5	60		229		293	332	
**Micro-Jet Gas Pressure** **(MPa)**	**Micro-Jet Stream Diameter** **(µm)**	**HAZ from the Steel Side S355J2** **(MPa)**	**Weld** **(MPa)**	**HAZ from the Alloy 59 Side (MPa)**	**UTS from the Steel Side S355JR** **(MPa)**	**UTS Weld** **(MPa)**	**UTS for HAZ from the Alloy 59 Side** **(MPa)**
without	without	211	276	319	745	974	1126
0.4	50	226	287	325	798	1013	1147
0.4	60	231	293	336	815	1034	1186
0.5	50	232	295	339	819	1041	1197
0.5	60	222	288	326	784	1017	1151

## Data Availability

Not applicable.
